# Latent Membrane Protein 1 Promotes Tumorigenesis Through Upregulation of PGC1β Signaling Pathway

**DOI:** 10.1007/s12015-020-10112-8

**Published:** 2021-01-09

**Authors:** Jia Feng, Qi Chen, Ping Zhang, Xiaodong Huang, Weiguo Xie, Hongyu Zhang, Paul Yao

**Affiliations:** 1 Department of Hematology, Peking University Shenzhen Hospital, 518036 Shenzhen, People’s Republic of China; 2 Institute of Rehabilitation Center, Tongren Hospital of Wuhan University, 430060 Wuhan, People’s Republic of China

**Keywords:** Hexokinase domain containing 1, Latent membrane protein 1, Nuclear factor-κB, 8-oxoguanine DNA glycosylase, Peroxisome proliferator‐activated receptor-γ coactivator 1β

## Abstract

Natural killer/T-cell lymphoma (NKTCL) is an aggressive Epstein-Barr virus (EBV)-associated non-Hodgkin lymphoma with poor prognosis. In this study, we aimed to investigate the potential mechanism of latent membrane protein 1 (LMP1)-mediated tumorigenesis and provide a novel therapeutic strategy for targeting the EBV DNA genome. We found that LMP1 upregulated the expression of peroxisome proliferator-activated receptor-γ (PPARγ) coactivator-1β (PGC1β) through activation of nuclear factor-κB (NF-κB). Furthermore, the activated PGC1β upregulated the expression of 8-oxoguanine DNA glycosylase (OGG1) through the coactivation of nuclear respiratory factor 1 (NRF1) and GA-binding protein α (GABPα), preventing reactive oxygen species (ROS)-mediated base incision in the EBV genome and favoring its survival. Interruption of hexokinase domain component 1 (HKDC1) by either shRNA or Tf-D-HKC8 peptide suppressed the interaction of HKDC1 with voltage-dependent anion channel 1 (VDAC1), triggering mitochondrial dysfunction and excessive generation of ROS, thus resulting in EBV suppression through ROS-mediated DNA damage. Suppression of the EBV genome inhibited the expression of the LMP1/PGC1β/HKDC1/OGG1 signaling pathway, forming a positive feed forward loop for the generation of ROS, hence inhibiting the EBV genome and subsequent EBV-associated tumor development. We concluded that LMP1 triggers EBV-associated tumorigenesis through activation of the PGC1β pathway. This study provided a novel therapeutic strategy for the treatment of EBV-associated tumors by targeting HKDC1.

**Supplementary Information:**

The online version contains supplementary material available at 10.1007/s12015-020-10112-8.

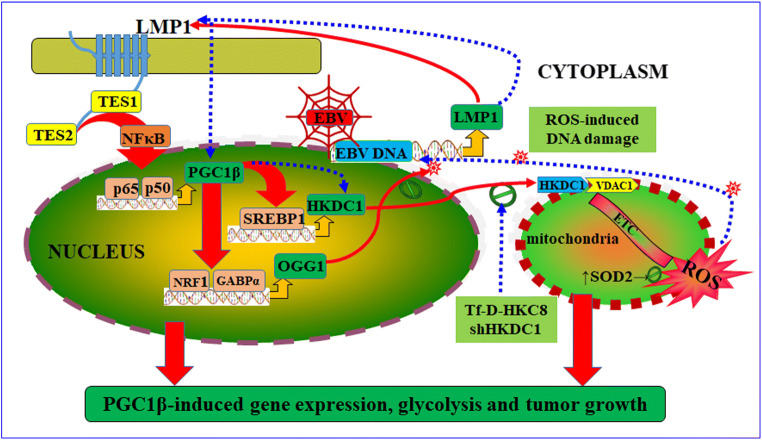

## Introduction

Natural killer/T-cell lymphoma (NKTCL) is an aggressive Epstein-Barr virus (EBV)-associated non-Hodgkin lymphoma with a high prevalence in most Asian populations [1–3]. Multiple pathogenic pathways have been reported to be involved in NKTCL [4]. Many different therapeutic targets, including programmed cell death 1 (PD-1) and P-glycoprotein (P-gp), have been extensively investigated and their targeting was shown to result in significant improvements in symptoms [5–7].Whereas, many cases were demonstrated to eventually relapse, resulting in poor prognosis [8, 9]. Many studies have shown that infection with EBV might be a potential driving force for tumor development, such as through oxidative stress-mediated EBV DNA damage, thus indicating EBV DNA as a novel therapeutic target in EBV-associated tumors [10, 11].

The latent membrane protein 1 (LMP1) encoded by EBV is known to be a direct target gene of Epstein–Barr virus nuclear antigen 2 (EBNA2). As an oncogene [12], LMP1 has been reported to act as a master metabolic regulator in many EBV-associated tumor cells by potentiating aerobic glycolysis, a condition known as the Warburg effect [13]. Briefly, LMP1 has 6 hydrophobic membrane-spanning domains and a 200-amino acid cytoplasmic carboxyl terminus, which is comprised of 2 domains, named transformation effector sites 1 and 2 (TES1 and TES2), respectively. These 2 domains are known to be responsible for the transformation of B-cells and the activation of the nuclear factor-κB (NF-κB) [14], which then translocates into the nucleus to activate its target genes [15]. It has been reported that activated NF-κB prevents cell apoptosis [16] and favors tumorigenesis. However, the detailed mechanisms underlying this effect remain largely unknown [17].

The peroxisome proliferator-activated receptor-γ (PPARγ) coactivator-1 (PGC1) family, which includes members such as PGC1α and PGC1β [18], has been reported to play an important role in maintaining the energy balance through the metabolism of glucose and lipids. More specifically, PGC1α and PGC1β have been shown to function as transcriptional coactivators regulating many downstream target genes [19]. It has been recently reported that PGC1β is critical in tumorigenesis through the regulation of mitochondrial biogenesis, glycolysis metabolism [20–23], and oxidative stress [24]. We recently found that PGC1β regulates tumor growth and metastasis through a lactate dehydrogenase A (LDHA)-mediated glycolytic metabolism [25], as well as through the expression of SREBP1-mediated hexokinase domain component 1 (HKDC1) [26]. Hexokinase (HK) is a rate-limiting enzyme known to phosphorylate hexose sugars to maintain the glucose metabolism [27]. Additionally, it has been shown to interact with the voltage-dependent anion channel (VDAC) protein, thus regulating the mitochondrial membrane potential (MMP) and apoptosis [28]. It has also been reported to be involved in tumor initiation and maintenance [29]. Similarly, HKDC1, another novel HK isoform, has been recently found to play a potential role in tumorigenesis through glucose homeostasis and oxidative stress [26, 30–33]. In addition, we recently found that HKDC1 C-terminal based peptides inhibited NKTCL by modulating the mitochondrial function and suppressing EBV [34]; however, the detailed mechanism remains unclear.

In this study, we aimed to investigate the potential mechanism of LMP1 protein-mediated tumorigenesis and provide a novel therapeutic strategy for targeting EBV DNA in NKTCL cells. We found that the expression of PGC1β was upregulated by the LMP1 protein through the activation of NF-κB on the PGC1β promoter. Furthermore, PGC1β was demonstrated to upregulate the expression of 8-oxoguanine DNA glycosylase (OGG1), an enzyme involved in base excision repair following DNA damage [35], through the coactivation of nuclear respiratory factor 1 (NRF1) and GA binding protein α (GABPα) binding elements on the OGG1 promoter, thus preventing EBV from oxidative stress-mediated DNA damage and favoring EBV survival. We also found that interruption of HKDC1 through either gene knockdown or HKDC1 C-terminal based peptides [36] inhibited its ability to bind with VDAC1, triggering mitochondrial dysfunction and the generation of reactive oxygen species (ROS), resulting in EBV DNA damage. This further triggered the suppression of the LMP1/PGC1β/HKDC1/OGG1 signaling pathway, providing a positive feedforward loop for the generation of ROS and suppression of the EBV DNA and EBV-mediated tumorigenesis in NKTCL cells. We concluded that the expression of LMP1 promoted tumorigenesis through the upregulation of the PGC1β/HKDC1/OGG1 signaling pathway. Thus, the interruption of HKDC1 might be a potential novel therapeutic strategy for the treatment of EBV-associated tumors.

## Results

### Expression of EBV LMP1 Protein Regulated the PGC1β/OGG1 Signaling Pathway and NF-κB Activity in NKTCL Cells

**Fig. 1:**
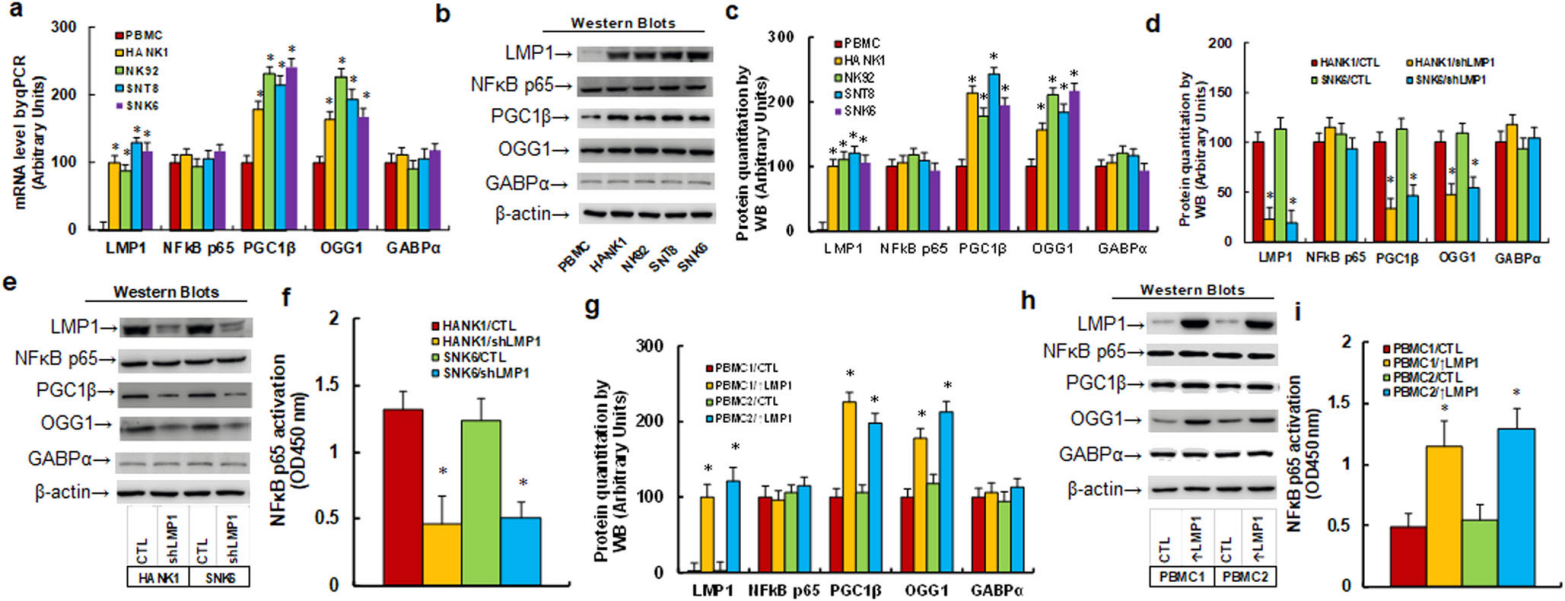
Expression of EBV LMP1 protein regulates the PGC1β/OGG1 signaling pathway and NF-κB activity in NKTCL cells. **a** Different NKTCL cell lines, including PBMC, HANK1, NK92, SNT8 and SNK6 cells, were used for mRNA analysis, n = 4. *, *P* < 0.05, vs. PBMC group. **b** Representative pictures for Western Blotting. **c** Protein quantitation for (**b**), n = 5. *, *P* < 0.05, vs. PBMC group. **d**-**f** The LMP1 was knockdown by lentivirus in HANK1 and SNK6 cells for analysis. **d** Protein quantitation for Western Blotting, n = 5; **e** Representative pictures for (**d**); **f** NF-κB p65 activation assay, n = 5; *, *P* < 0.05, vs. HANK1/CTL. **g**-**i **The LMP1 was overexpressed by lentivirus in PBMCs for analysis. **g** Protein quantitation for Western Blotting, n = 5; **h** Representative pictures for (**g**); **i** NF-κB p65 activation assay, n = 5; *, *P* < 0.05, vs. PBMC1/CTL. Data were expressed as mean ± SEM

We first evaluated the gene expression of the PGC1β/OGG1 signaling pathway in different NKTCL cell lines, including PBMC, HANK1, NK92, SNT8, and SNK6 cells. Our results showed that the LMP1 mRNA was not expressed in healthy PBMCs, whereas significant levels of expression were observed in other NKTCL cell lines. Additionally, we found that the mRNA levels of PGC1β and OGG1 were increased to 178% and 164% in HANK1 cells compared with PMBC cells. We noted that other NKTCL cell lines had significantly increased expression levels similar to those observed in HANK1 cells; however, the expression levels of the NF-κB subunit p65 and GABPα did not show a significant difference (Fig. 1a). We also evaluated the gene expression of healthy Natural killer (NK) and PBMC cells for those genes, and the results showed no significant difference (Fig. S1). We then measured the protein levels for those genes, and detected an expression pattern similar to that of the mRNA levels (Fig. 1b and c, S2a). Next, we evaluated the potential effect of a LMP1 knockdown (shLMP1) in NKTCL cells. Our results showed that the levels of the LMP1 protein were decreased to 23% and 19% in HANK1 and SNK6 cells, respectively. The expression of PGC1β was also shown to be decreased to 34% and 46%, while the expression of OGG1 was decreased to 47% and 54%, respectively, compared with that in the HANK1/CTL group. Whereas the expression of the NF-κB subunit p65 and GABPα showed no difference (Fig. 1d and e, S2b). We also measured the activity of NF-κB p65. Our results showed that following a LMP1 knockdown in HANK1 and SNK6 cells, the activity of NF-κB p65 was decreased to 35% and 39%, respectively, compared with the HANK1/CTL group (Fig. 1f). Finally, we evaluated the potential effect of the expression of LMP1 in healthy PBMCs and found that following infection with a LMP1-expressing lentivirus the LMP1 protein was significantly expressed in 2 lines of PBMCs. In addition, the expression of PGC1β was demonstrated to be increased to 226% and 198%, while the expression of OGG1 was increased to 178% and 213%, compared with the PBMC1/CTL group. Again, we noted that the expression of the NF-κB subunit p65 and GABPα showed no difference (Fig. 1g, h, S2c). We also measured the activity of NF-κB p65 following the expression of LMP1 in PBMC1 and PBMC2 cells, and found that it was increased to 235% and 263%, respectively, compared with the PBMC1/CTL group (Fig. 1i). Our results suggested that the expression of PGC1β and OGG1 in addition to the activity of NF-κB were regulated by the expression of LMP1 in NKTCL cells.

### Expression of PGC1β was Regulated By LMP1-mediated Activation of NF-κB on the PGC1β Promoter

**Fig. 2:**
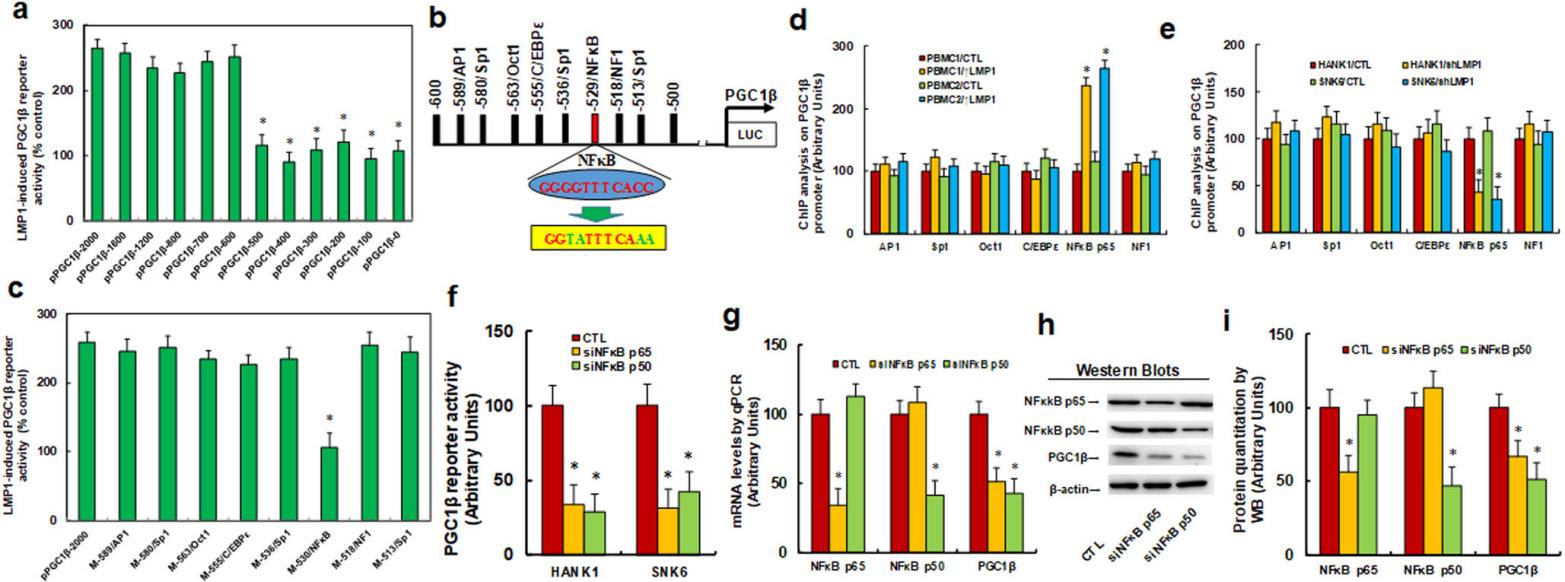
PGC1β expression is regulated by LMP1-mediated NF-κB activation on the PGC1β promoter. **a** The conditionally immortalized PBMCs were infected by either LMP1 or empty control (CTL) lentivirus for 2 days, and then the cells were transiently transfected by either PGC1β full length (pPGC1β-2000) or deletion reporter plasmids. After 24 hours, the LMP1-induced PGC1β reporter activities were calculated as the relative percentage (% control) in comparison to lentivirus empty control (CTL) infected cells. *, *P* < 0.05, vs. pPGC1β-2000 group, n = 5. **b** The schematic picture for the potential transcriptional binding motif in the range of -600~-500 (from transcription start site) on the PGC1β promoter in addition to the potential NF-κB binding site are marked in red, and the related mutation sites are marked in green. **c** The cells were transiently transfected by either the PGC1β full length (pPGC1β-2000) or the specific transcriptional binding motif mutation reporter plasmids, and then the reporter activities were measured after 24 hours. *, *P* < 0.05, vs. pPGC1β-2000 group, n = 5. **d** Immortalized PBMCs were infected by LMP1 lentivirus and then cells were used for ChIP analysis using different antibodies, and the PGC1β promoter was amplified and measured by qPCR, n = 5. *, *P* < 0.05, vs. PBMC1/CTL group. **e** LMP1 was knockdown by lentivirus in HANK1 and SNK6 cells for ChIP analysis on PGC1β promoter, n = 5. *, *P* < 0.05, vs. HANK1/CTL group. **f**-**i** The siRNA was used to knockdown either NF-κB p65 or p50 in HANK1 and SNK6 cells for analysis; **f** PGC1β reporter activity; **g** mRNA levels by qPCR; **h** Representative pictures for Western Blotting, n = 5; **i** Protein quantitation for (**h**). n = 5. *, *P* < 0.05, vs. CTL group. Data were expressed as mean ± SEM

We investigated the possible molecular mechanism underlying the LMP1-mediated PGC1β activation. We initially generated a series of progressive 5′-promoter deletion constructs for the PGC1β promoter, and transfected them into conditional immortalized PBMCs infected with either LMP1 or empty control lentivirus for the analysis of the activity of the PGC1β reporter. Our results showed that the LMP1-induced activation of the PGC1β reporter was not altered among the − 2000, -1600, -1200, -800, -700, and − 600 deletion constructs (numbered according to Ensembl gene ID: PPARGC1B-201 ENST00000309241.9; transcription start site was marked as 0), whereas the LMP1-induced activation was shown to disappear in the − 500 deletion construct and subsequent deletion reporter constructs. These observations indicated that the LMP1-responsive transcriptional element was located in the range of -600 to approximately − 500 on the PGC1β promoter (Fig. 2a). Analysis of the transcription factor databases revealed the existence of many potential binding motifs, including those for AP1, Sp1, Oct1, C/EBPε, NF1, and NF-κB (marked in red) located in the range of -600 to -500 on the PGC1β promoter (Fig. 2b). We then mutated all of these potential binding motifs on the full length PGC1β reporter construct (pPGC1β-2000), and found that the LMP1-induced activation of the reporter disappeared only in the case of the NF-κB mutation construct (from ggggtttcacc to ggtatttcaaa, located at -529, marked in green, Fig. 2b). This finding indicated that LMP1 induced the activation of PGC1β through the NF-κB binding motif on the PGC1β promoter (Fig. 2c). We then conducted ChIP analysis using antibodies for potential transcription factors located in the range of -600 to -500. We first infected 2 PBMC lines with a LMP1-expressing lentivirus and observed that only NF-κB p65 exhibited an increased binding ability (Fig. 2d). Furthermore, we knocked-down LMP1 in 2 NKTCL cell lines, HANK1 and SNK6, using shLMP1, and found that among all transcription factors only NF-κB p65 had a decreased binding ability on the PGC1β promoter (Fig. 2e). We also evaluated the potential effects of knocking-down NF-κB. We accordingly observed that the PGC1β luciferase reporter activity was significantly decreased in both HANK1 and SNK6 cells transected with either siNF-κB-p65 or siNF-κB-p50 (Fig. 2f). We also evaluated the gene expression in these constructs. Our results showed that the levels of expression of both mRNA and protein of NF-κB-p65 and NF-κB-p50 were decreased to approximately 50% in SNK6 cells following transfection with these siRNAs, indicating successful gene knockdown. Furthermore, the levels of expression of both PGC1β mRNA (Fig. 2g) and protein (Fig. 2h and i, and FS3) were demonstrated to exhibit significant decreases as a result of the siRNA treatment for both p65 and p50 subunits. Our results indicated that the expression of PGC1βwas regulated by the LMP1-mediated activation of NF-κB on the PGC1β promoter.

### Expression of OGG1 was Regulated By PGC1β-mediated Activation of NRF1/GABPα on the OGG1 Promoter

**Fig. 3:**
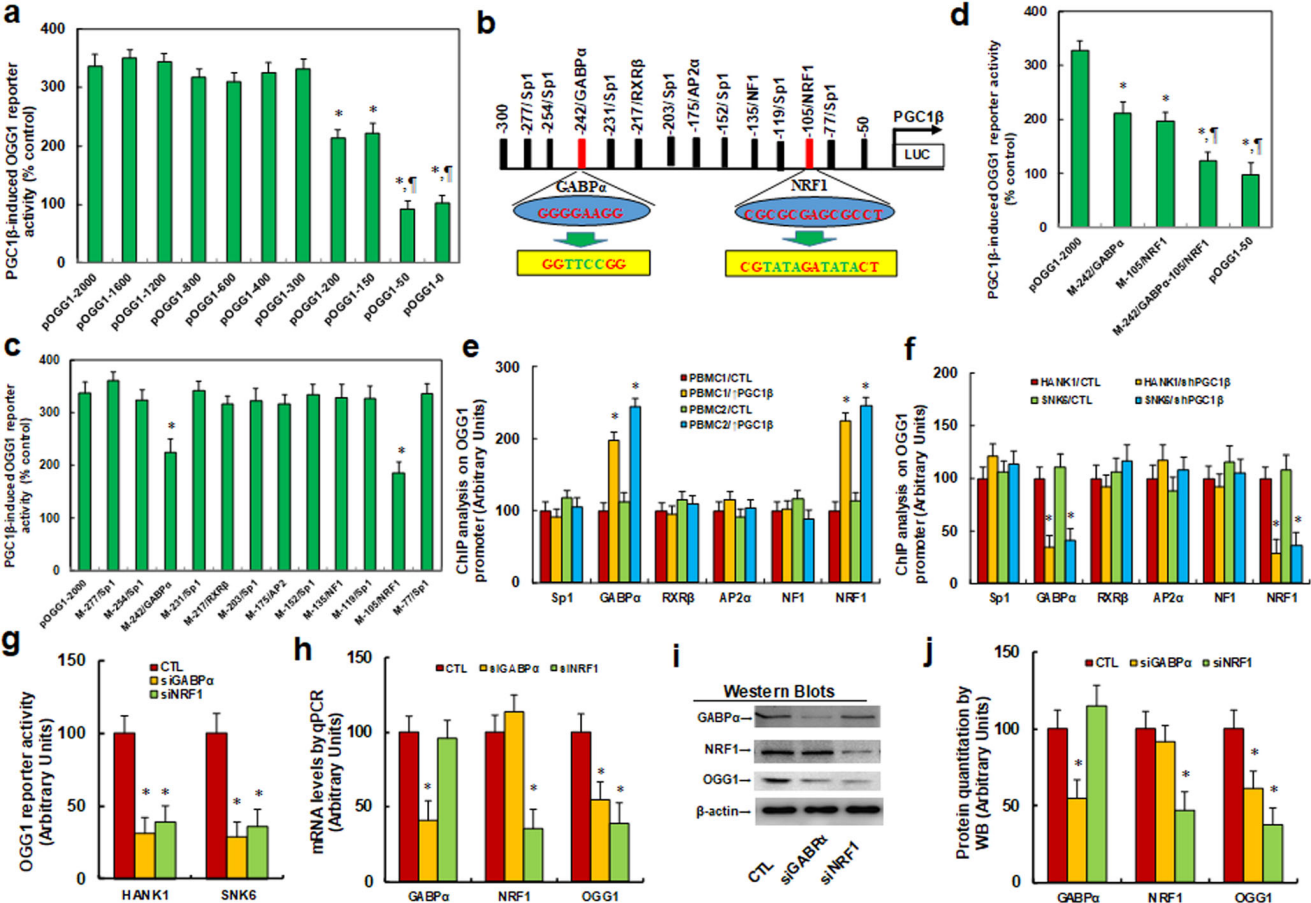
OGG1 expression is regulated by PGC1β-mediated NRF1/GABPα activation on the OGG1 promoter. **a** The conditionally immortalized PBMCs were infected by either PGC1β or empty control (CTL) lentivirus for 2 days, and then the cells were transiently transfected by either OGG1 full length (pOGG1-2000) or deletion reporter plasmids. After 24 hours, the PGC1β -induced OGG1 reporter activities were calculated as the relative percentage (% control) in comparison to lentivirus empty control (CTL) infected cells. *, *P* < 0.05, vs. pOGG1-2000 group, n = 5. **b** The schematic picture for the potential transcriptional binding motif in the range of -300~-50 (from transcription start site) on the OGG1 promoter; the potential binding sites for NRF1 and GABPα are marked in red, and the related mutation sites are marked in green. **c** The cells were transiently transfected by either OGG1 full length (pOGG1-2000) or the specific transcriptional binding motif mutation reporter plasmids, and then the reporter activities were measured after 24 hours. *, *P* < 0.05, vs. pOGG1-2000 group, n = 5. **d** The cells were transiently transfected by either OGG1 full length (pOGG1-2000), single mutant, or double mutants for NRF1 and GABPα, and then the reporter activities were measured after 24 hours. *, *P* < 0.05, vs. pOGG1-2000 group, n = 5. **e** Immortalized PBMCs were infected by PGC1β lentivirus; then, cells were used for ChIP analysis using different antibodies, and the PGC1β promoter was amplified and measured by qPCR, n = 5. *, *P* < 0.05, vs. PBMC1/CTL group. **f** PGC1β was knockdown by lentivirus in HANK1 and SNK6 cells for ChIP analysis on PGC1β promoter, n = 5. *, *P* < 0.05, vs. HANK1/CTL group. **g**-**j** The siRNA was used to knockdown either GABPα or NRF1 in HANK1 and SNK6 cells for analysis; **g** OGG1 reporter activity; **h** mRNA levels by qPCR; **i **Representative pictures for Western Blotting, n = 5; **j** Protein quantitation for (**i**). n = 5. *, *P* < 0.05, vs. CTL group. Data were expressed as mean ± SEM

 We also investigated the potential mechanism of the PGC1β-mediated activation of the expression of OGG1. We generated a series of progressive 5′-promoter deletion constructs for the OGG1 promoter, and transfected these constructs into conditional immortalized PBMCs infected with either PGC1β or empty control lentivirus for the analysis of the activity of the OGG1 reporter. We respectively found that the PGC1β-induced activation of the OGG1 reporter was not altered among the − 2000, -1600, -1200, -800, -600, and − 400, and − 300 deletion constructs (numbered according to the Ensembl gene ID: OGG1-201 ENST00000302003.11; transcription start site was marked as 0), whereas the PGC1β-induced activation was demonstrated to be partly decreased in the − 200 and − 150 deletion constructs, and completely disappeared in the − 50 and 0 deletion constructs. These findings indicated that the PGC1β-responsive transcriptional element was located in the range of -300 to approximately − 50 on the OGG1 promoter (Fig. 3a). Moreover, analysis of transcription factor databases revealed the presence of many potential binding motifs, including the Sp1, GABPα (marked in red), RXRβ, AP2α, NF1, and NRF1 (marked in red) binding sites located in the range of -300 to -50 on the OGG1 promoter (Fig. 3b). We then mutated all of these potential binding motifs on the full length OGG1 reporter construct (pOGG1-2000) and found that the PGC1β1-induced activation of the reporter was significantly decreased in both GABPα (from ggggaagg to ggttccgg, in green) and NRF1 (from cgcgcgagcgcct to cgtatagatatact, in green) mutation constructs (Fig. 3b). This finding indicated that PGC1β induced the activation of OGG1 through GABPα (located at -242) and NRF1 (located at -105) binding motifs on the OGG1 promoter (Fig. 3c). We then generated single mutations, as well as a double mutation on the GABPα and NRF1 binding sites in the pOGG1 full-length construct (M-242/GABPα-105/NRF1) and found that whereas each single mutant led to the partial decrease in the activation of OGG1, the double mutant completely diminished the PGC1β-induced activation of OGG1 (Fig. 3d). We then conducted ChIP analysis using antibodies for potential transcription factors located in the range of -300 to -50. To this end, we first infected 2 PBMC lines with a PGC1-expressing lentivirus and observed that both GABPα and NRF1 exhibited an increased binding ability (Fig. 3e). Furthermore, we knocked-down PGC1β in 2 NKTCL cell lines, HANK1 and SNK6, using shPGC1β, and respectively found that both GABPα and NRF1 exhibited decreased binding ability on the OGG1 promoter (Fig. 3f). We also evaluated the potential effect of knocking-down either the GABPα or NRF1 gene. We noted that the OGG1 luciferase reporter activity was significantly decreased as a result of treatment of both HANK1 and SNK6 cells with either siGABPα or siNRF1 (Fig. 3g). We also evaluated the levels of gene expression following treatment with these siRNAs and noted that the levels of both the mRNA and protein expression of both GABPα and NRF1 were significantly decreased in SNK6 cells, indicating successful gene knockdown. Furthermore, we observed that the levels of expression of both OGG1 mRNA (Fig. 3h) and protein (Fig. 3i and j and FS4) exhibited a significant decrease as a result of the siRNA treatment for both GABPα and NRF1. Our results thus indicated that the expression of OGG1 was regulated by the PGC1β-mediated activation of either or both GABPα and NRF1 on the PGC1β promoter.

### Tf-D-HKC8 Peptide‐mediated Interruption of HKDC1 Induced Suppression of PGC1β/OGG1 and Mitochondrial Dysfunction

**Fig. 4:**
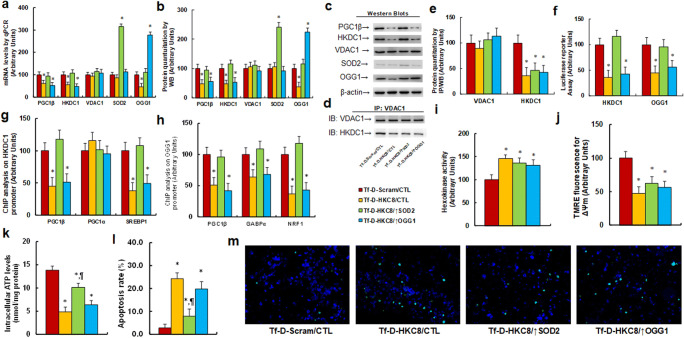
Tf-D-HKC8 peptide-mediated HKDC1 interruption induces PGC1β/OGG1 suppression and mitochondrial dysfunction. SNK6 cells were infected by SOD2, OGG1 or control (CTL) lentivirus and then treated with either scramble (Scram) or D-Tf-HKC8 peptide (0.5 µM) for two days before being harvested for biomedical analysis. **a**) mRNA levels by qPCR, n = 4. (b) Protein quantitation for Western Blotting. (c) Representative pictures for (b), n = 5. (d) Representative pictures for IP/WB. (e) Protein quantitation for (d), n = 5. (f) Luciferase reporter activities for PGC1β and OGG1, n = 5. (g) ChIP analysis on HKDC1 promoter, n = 4. (h) ChIP analysis on OGG1 promoter, n = 4. (i). HKDC1 activity assay, n = 5. (j) Mitochondrial membrane potential (∆Ψm), n = 5. (k) Intracellular ATP level, n = 5. (l) Apoptosis rate by TUNEL assay, n = 5. (m) Representative pictures for (l). *, *P* < 0.05, vs. Tf-D-Scram/CTL group; ¶, *P* < 0.05, vs. Tf-D-HKC8/CTL group. Data were expressed as mean ± SEM

We evaluated the potential effect of the Tf-D-HKC8 peptide on the PGC1β/OGG1 signaling pathway and mitochondrial function in SNK6 cells. We first evaluated the gene expression of the PGC1β/OGG1 signaling pathway. Our results showed that treatment with Tf-D-HKC8 (Tf-D-HKC8/CTL) significantly decreased the mRNA levels of PGC1β, HDKC1, and OGG1. Conversely, infection with a SOD2-expressing lentivirus (Tf-D-HKC8/↑SOD2) completely reversed this effect, whereas infection with an OGG1-expressing lentivirus (Tf-D-HKC8/↑OGG1) had little effect, compared with the control group (Tf-D-Scram/CTL). In addition, we observed that the VDAC1 mRNA levels did not change as a result of any of these treatments, while overexpression of SOD2 and OGG1 was shown to significantly increase each of the related mRNA levels, indicating successful infection (Fig. 4a). We then measured the protein levels for those genes, and found an expression pattern similar to that of the mRNA levels (Fig. 4b and c, S5a). Next, we evaluated the interaction between VDAC1 and HKDC1 using immunoprecipitation (IP)/western blotting (WB) techniques. Our results showed that treatment with Tf-D-HKC8/CTL significantly interrupted the binding ability of HKDC1 with VDAC1, reducing it to 36% compared with the Tf-D-Scram/CTL group, whereas overexpression of either SOD2 or OGG1 had no effect (Fig. 4d and e, S5b). We then evaluated the effect of the Tf-D-HKC8 peptide on reporter activities and found that treatment with Tf-D-HKC8/CTL significantly decreased the luciferase reporter activities of both PGC1β and OGG1, with overexpression of SOD2 completely reversing this effect, whereas overexpression of OGG1 had no effect (Fig. 4f). We also evaluated the effect of the Tf-D-HKC8 peptide on the binding ability of transcription factors on promoters. Our results showed that treatment with Tf-D-HKC8/CTL significantly decreased the ability of PGC1β and SREBP1 to bind to the HKDC1 promoter, whereas we did not observe any effect on the binding ability of PGC1α (Fig. 4g). Additionally, treatment with Tf-D-HKC8/CTL was demonstrated to significantly decrease the ability of PGC1β, GABPα, and NRF1 to bind to the OGG1 promoter (Fig. 4h). Again, we noted that overexpression of SOD2 completely reversed this effect, whereas overexpression of OGG1 had no effect. We also evaluated the changes in the activity of hexokinase as a result of different treatments. We accordingly found that treatment with Tf-D-HKC8/CTL increased the levels of the activity of hexokinase to 146% compared with the Tf-D-Scram/CTL group, whereas little effect was noted following infection with either SOD2 or OGG1, indicating successful peptide treatment and subsequent cellular internalization (Fig. 4i). Finally, we evaluated the effect of the Tf-D-HKC8 peptide on mitochondrial function and observed that treatment with Tf-D-HKC8/CTL decreased the mitochondrial membrane potential (MMP, ΔΨm) to 47%, whereas no such effect was observed by the overexpression of either SOD2 or OGG1 (Fig. 4j). Furthermore, we found that the intercellular ATP levels were decreased to 35%. Conversely, overexpression of SOD2 completely reversed this effect, whereas overexpression of OGG1 had no effect (Fig. 4k). In addition, treatment with Tf-D-HKC8/CTL increased the apoptosis rate to 868%, with overexpression of SOD2 partly reversing this effect, whereas overexpression of OGG1 had no effect (Fig. 4l, m). Our results indicated that the Tf-D-HKC8 peptide-mediated interruption of HKDC1 induced the suppression of PGC1β/OGG1 and mitochondrial dysfunction. This effect was completely and partly reversed with the overexpression of SOD2 and OGG1, respectively.

### Tf-D-HKC8 Peptide‐mediated Interruption of HKDC1 Induced Oxidative Stress and Suppression of EBV

**Fig. 5:**
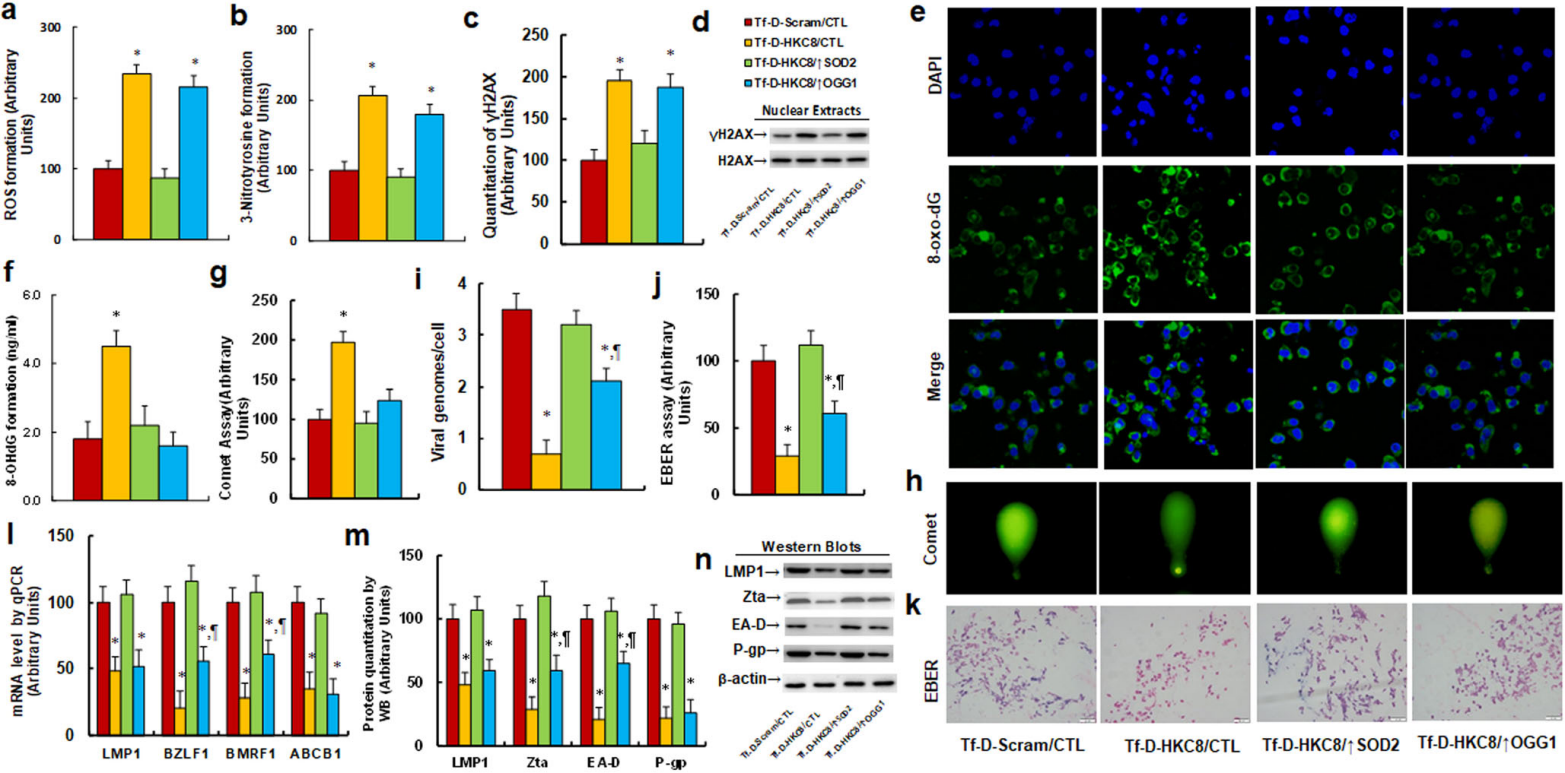
Tf-D-HKC8 peptide-mediated HKDC1 interruption induces oxidative stress and EBV suppression. SNK6 cells were infected by SOD2, OGG1 or control (CTL) lentivirus and then treated with either scramble (Scram) or D-Tf-HKC8 peptide (0.5 µM) for two days before being harvested for biomedical analysis. **a** ROS formation, n = 5. **b** 3-nitrotyrosine (3-NT) formation, n = 5. **c** Quantitation of γH2AX formation, n = 5. **d** Representative pictures for (**c**). **e** Representative pictures for 8-oxo-dG staining. **f** 8-OHdG formation, n = 5. **g** Quantitation of comet assay, n = 4. **h** Representative pictures for (**g**). **i** EBV viral genomes/cell by qPCR, n = 4. **j** Quantitation for EBER assay, n = 4. **k** Representative pictures for (**j**). **l **mRNA level by qPCR for BZLF1, BMRF1 and ABCB1, n = 4. **m** Protein quantitation for Zta, EA-D and P-gp, n = 5. **n** Representative pictures for (**m**). *, *P* < 0.05, vs. Tf-D-Scram/CTL group; ¶, *P* < 0.05, vs. Tf-D-HKC8/CTL group. Data were expressed as mean ± SEM

We first evaluated the effect of SOD2 on Tf-D-HKC8 peptide-induced oxidative stress. Our results showed that treatment with Tf-D-HKC8/CTL increased the generation of ROS to 234% (Fig. 5a) and the formation of 3-nitrotyrosine to 207% (Fig. 5b), compared with the Tf-D-Scram/CTL group. Interestingly, we noted that overexpression of SOD2 completely reversed this effect, whereas overexpression of OGG1 had no effect. We then evaluated the effect of SOD2 on Tf-D-HKC8 peptide-induced DNA damage and found that treatment with Tf-D-HKC8/CTL increased the formation of γH2AX to 195%, with overexpression of SOD2 but not OGG1 completely reversing this effect (Fig. 5c and d, S6a). In addition, treatment with Tf-D-HKC8/CTL was demonstrated to increase the formation of 8-OHdG to 250% (Fig. 5e and f) and the tail length in the comet assay to 197% (Fig. 5g, h), with overexpression of both SOD2 and OGG1 completely reversing this effect. We also evaluated the effect of the Tf-D-HKC8 peptide on the replication of EBV. We observed that treatment with Tf-D-HKC8/CTL decreased the amount of EBV genomic DNA copies to 20% (Fig. 5i) and decreased the EBV-encoded RNA to 29%, as indicated by an EBER assay (Fig. 5j, k), compared with the Tf-D-Scram/CTL group. Overexpression of SOD2 and OGG1 reversed this effect completely and partly, respectively. Finally, we evaluated the effect of the Tf-D-HKC8 peptide on EBV-related gene expression. Our results showed that treatment with Tf-D-HKC8/CTL significantly decreased the mRNA levels of LMP1, BZLF1, BMRF1, and ABCB1 compared with those in the Tf-D-Scram/CTL group. Conversely, overexpression of SOD2 was shown to completely reverse this effect, whereas overexpression of OGG1 partly restored the expression of BZLF1 and BMRF1, without affecting the expression of LMP1 or ABCB1 (Fig. 5l). We then measured the protein levels of these genes and obtained an expression pattern similar to that of the mRNA levels (Fig. 5m and n, S6b). Our results indicated that Tf-D-HKC8 peptide-mediated interruption of HKDC1 induced oxidative stress and suppression of EBV, which was completely and partly reversed by overexpression of SOD2 and OGG1, respectively.

### Tf-D-HKC8 Peptide‐mediated Interruption of HKDC1 Suppressed Tumor Growth

**Fig. 6:**
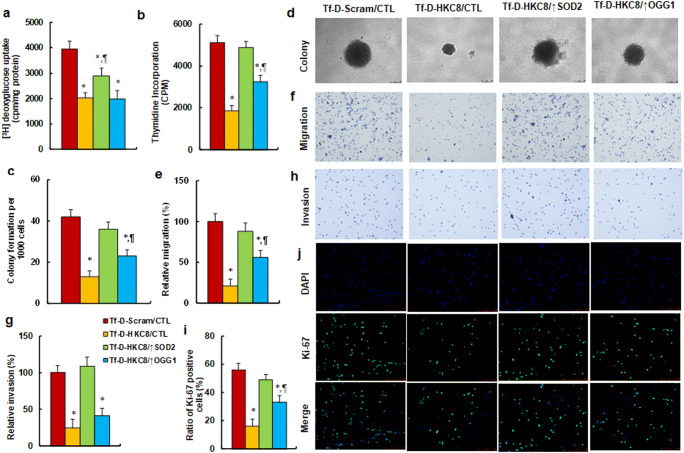
Tf-D-HKC8 peptide-mediated HKDC1 interruption suppresses tumor growth. SNK6 cells were infected by SOD2, OGG1 or control (CTL) lentivirus and were then treated with either scramble (Scram) or D-Tf-HKC8 peptide (0.5 µM) for 2 days before being harvested for biomedical analysis. **a** [^3^H]deoxyglucose uptake, n = 4. **b** Cell proliferation analysis by thymidine incorporation, n = 5. **c** Colony formation assay in soft agar, n = 4. **d** Representative pictures for colony formation. **e** Cell migration assay, n = 4. **f** Representative pictures for (**e**). **g** Cell invasion assay, n = 4. **h** Representative pictures for (**g**). **i** Quantitation of Ki-67 positive cells, n = 3. **j** Representative picture for Ki-67 staining. *, *P* < 0.05, vs. CTL group; ¶, *P* < 0.05, vs. Tf-D-HKC15 group. Data were expressed as mean ± SEM

We evaluated the effect of SOD2 on Tf-D-HKC8 peptide-mediated tumor suppression. We found that treatment with Tf-D-HKC8/CTL decreased deoxyglucose uptake to 51% (Fig. 6a) and thymidine incorporation to 36% (Fig. 6b) compared with the Tf-D-Scram/CTL group. Whereas, overexpression of either SOD2 or OGG1 was shown to partly reverse this effect. Likewise, treatment with Tf-D-HKC8/CTL decreased colony formation to 31% (Fig. 6c and d), cell migration to 21% (Fig. 6e and f), and the Ki-67 positive cell rate to 29% (Fig. 6i and j) with overexpression of SOD2 and OGG1 completely and partly, reversing this effect, respectively. In addition, we noted that treatment with Tf-D-HKC8/CTL decreased cell invasion to 25%; again, overexpression of SOD2 was demonstrated to completely reverse this effect, whereas overexpression of OGG1 had no effect on this process (Fig. 6g, h). Our results suggested that Tf-D-HKC8 peptide-mediated interruption of HKDC1 suppressed tumor growth with overexpression of SOD2 and OGG1 completely and partly reversing this effect, respectively.

### HKDC1 Knockdown Suppressed the PGC1β/OGG1 Signaling Pathway in Addition to EBV Replication and Tumor Growth

**Fig. 7:**
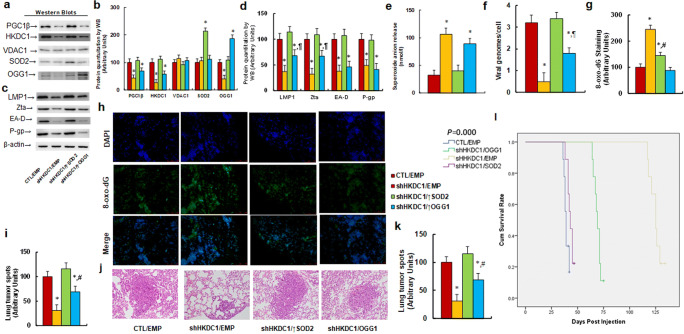
HKDC1 knockdown suppresses the PGC1β/OGG1 signaling pathway in addition to EBV replication and tumor growth. SNK6 cells were knocked down by either control (CTL) or HKDC1 through puromycin-selected lentivirus, and then overexpressed by empty (EMP), SOD2, or OGG1 through hygromycin-selected lentivirus for preparation of stable cell lines. The above 2 × 10^6^ of viable treated cells were injected through the tail vein for *in vivo* xenograft tumor development study in nude mice, and the treated mice were sacrificed for further analysis. **a**-**d** The tumor tissues from the lung were isolated for protein analysis, n = 4; **a** Representative bands for western blots. **b** Protein quantitation of PGC1β/OGG1 pathway for (**a**); **c** Representative bands for western blots. **d** Quantitation of EBV proteins for (**c**). **e** Superoxide anion release from tumor tissues, n = 5. **f** EBV viral genomes/cell by qPCR, n = 4. **g** Quantitation of 8-oxo-dG staining, n = 4. **h** Representative pictures for (**g**). **i**-**k** Mice were killed upon 20% weight loss, and the lungs were harvested for terminal analysis. Metastatic tumor nodules from the lungs were counted, and then the formalin-fixed, paraffin-embedded tumor tissue of the lung was sectioned to 4 mm thickness before histopathological analyses were performed with H&E staining. Images were taken using a Carl Zeiss MIRAX MIDI slide scanner, and the lung tumor spots were analyzed using a 3DHISTECH Pannoramic Viewer. **i** Tumor colony formation in lung, n = 9. **j** Representative picture by H&E staining. **k** Quantitated lung tumor spots, n = 5. **l** Kaplan-Meier analysis comparing survival of mice between each treatment group, *P* value represents log-rank Mantel-Cox test result, n = 9. *, *P* < 0.05, vs. CTL/EMP group; ¶, *P* < 0.05, vs. shHKDC1/EMP group. Data were expressed as mean ± SEM

We evaluated the effect of LMP1 on tumor growth through the activation of PGC1β and the HKDC1 interruption-mediated tumor suppression through the generation of ROS in a mouse model. We injected nude mice with treated SNK6 cells through the tail vein. We then isolated the generated xenograft tumor tissues from the lungs for analysis, and calculated the survival rate. We first measured the protein expression in the PGC1β/OGG1 signaling pathway in tumor tissues and found that treatment with shHKDC1 (shHKDC1/EMP) significantly decreased the levels of the PGC1β, HDKC1, and OGG1 proteins. Conversely, infection with a SOD2-expressing lentivirus (shHKDC1/↑SOD2) completely reversed this effect, whereas an OGG1-expressing lentivirus (shHKDC1/↑OGG1) had little effect, compared with the control group (CTL/EMP). Additionally, we observed that the levels of the VDAC1 protein were not altered under any of the treatments, whereas lentiviral infection with SOD2 and OGG1 significantly increased the levels of each protein, indicating successful lentiviral manipulation of gene expression (Fig. 7a and b, S7a). We then evaluated the effect of the interruption of HKDC1 on EBV-related protein expression. Our results showed that treatment with shHKDC1 significantly decreased the levels of the LMP1, Zta (coded by BZLF1), EA-D (coded by BMRF1), and P-gp (coded by ABCB1) proteins compared with those in the CTL/EMP group. In this case too, we observed that overexpression of SOD2 completely reversed this effect, whereas overexpression of OGG1 partly restored the expression of LMP1 and Zta, but had no effect on EA-D and P-gp (Fig. 7c and d, S7b). We then evaluated the release of superoxide anion (O_2_^−^.) from xenograft tumor tissues and found that treatment with shHKDC1/EMP increased O_2_^−^. release to 331% compared with the CTL/EMP group, with overexpression of SOD2 but not OGG1 completely reversing this effect (Fig. 7e). A similar effect was demonstrated on the amount of EBV genomic DNA copies, which were decreased to 16% (Fig. 7f). In addition, we noted that treatment with shHKDC1/EMP increased the formation of 8-oxo-dG to 245%, with overexpression of SOD2 partly, whereas OGG1 completely reversing this effect (Fig. 7g, h). Subsequently, we investigated the effect of treatments on tumor nodule formation and showed that treatment with shHKDC1/EMP decreased tumor colony formation in the lung to 32% (Fig. 7i) and lung tumor spots to 31%, as indicated by H&E staining (Fig. 7j, k) These effects were shown to be completely and partly reversed by overexpression of SOD2 and OGG1, respectively. Finally, we evaluated the effects of treatments on mouse survival rates using Kaplan-Meier analysis. Our results showed that treatment with shHKDC1/EMP significantly increased mouse survival rates compared with that for the CTL/EMP group. Once again, overexpression of SOD2 and OGG1 completely and partly diminished this effect, respectively (Fig. 7l). Our results suggested that knocking-down HKDC1 suppressed the PGC1β/OGG1 signaling pathway in addition to the replication of EBV and tumor growth in a mouse model. These effects were completely and partly reversed by the overexpression of SOD2 and OGG1, respectively.

###  Schematic Model of the EBV LMP1-induced PGC1β Signaling Pathway and Tumor Growth

**Fig. 8:**
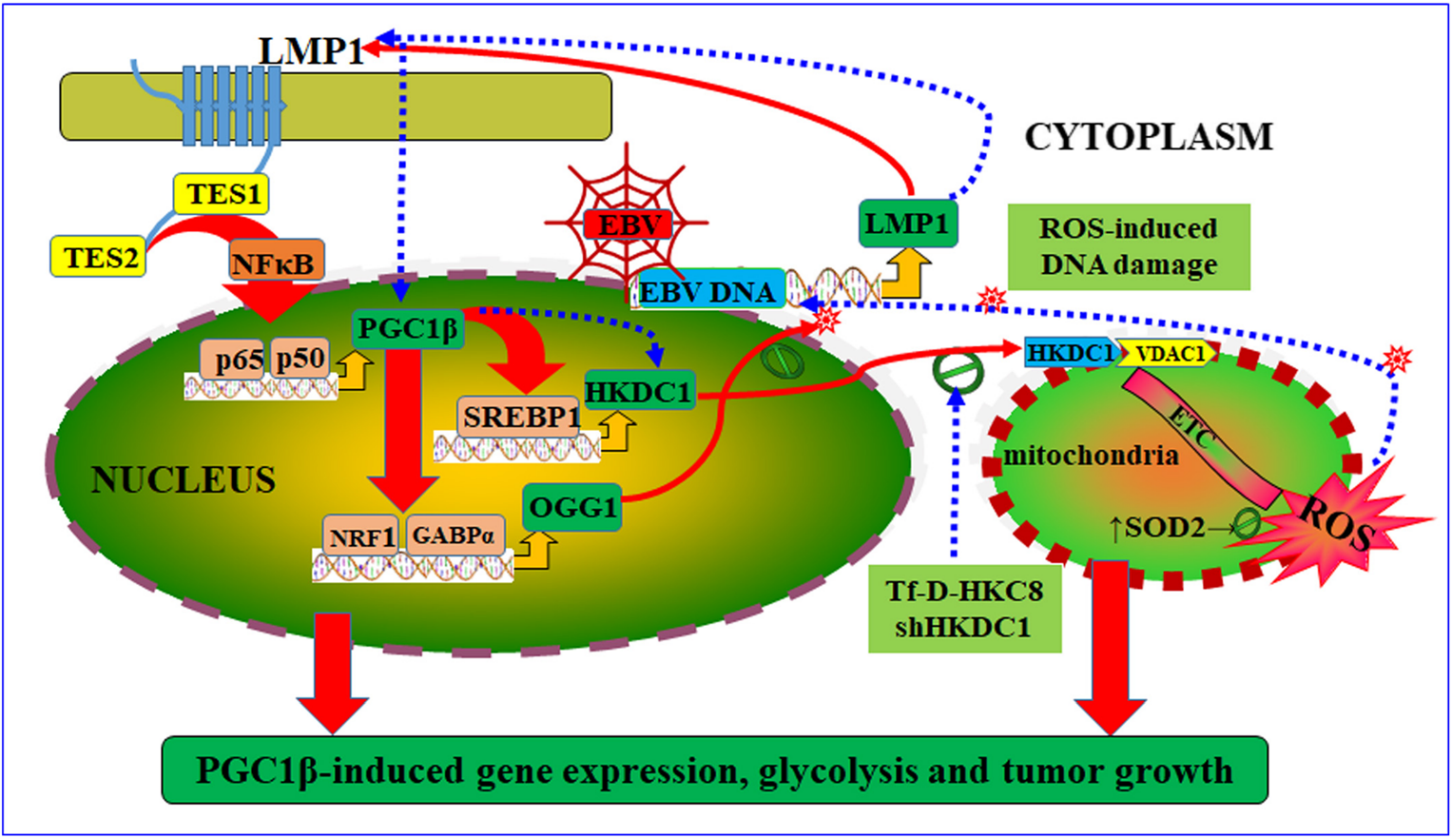
Schematic model for EBV LMP1-induced PGC1β signaling pathway and tumor growth. Abbreviations: EBV: Epstein-Barr virus; ETC: electron transport chain; GABPα: GA-binding protein α; HKDC1: Hexokinase domain component 1; LMP1: latent membrane protein 1; NRF1: (nuclear respiratory factor 1); NF-κB: nuclear factor-κB; OGG1: 8-oxoguanine DNA glycosylase 1; PGC1β: PGC1β: peroxisome proliferator-activated receptor-γ (PPARγ) coactivator-1β; ROS; reactive oxygen species; SOD2: superoxide dismutase 2; TES: transformation effector site; Tf-D-HKC8: last 8 amino acid of HKDC1 at the C-terminal (HKC8) with D-configuration using transferrin (Tf) receptor internalization sequence; VDAC1: vascular endothelial growth factor 1

We created a schematic model illustrating the LMP1-mediated tumor development through the activation of PGC1β and tumor suppression through the interruption of HKDC1 (Fig. 8). According to this model, the EBV-encoded LMP1 protein activates NF-κB, causing subunits p65 and p50 to bind to the NF-κB responsive element located on the PGC1β promoter, thus increasing the expression of PGC1β. Subsequently, the upregulated PGC1β further increases the expression of HKDC1 through the coactivation of SREBP1 and the expression of OGG1 through the coactivation of NRF1 and GABPα. The upregulated HKDC1 leads to increased mitochondrial function through its binding with VDAC1, favoring glycolysis and tumor growth. Concomitantly, the upregulation of OGG1 minimizes the generation of 8-oxo-dG, and increases DNA repair to prevent ROS-induced DNA damage in the EBV genome (shown in red circles). Whereas, interruption of HKDC1 through either the Tf-D-HKC8 peptide or shRNA inhibits the binding of HKDC1 and VDAC1, triggering mitochondrial dysfunction and generation of ROS. Increased generation of ROS results in direct EBV DNA damage with suppression of LMP1 and subsequent suppression of the PGC1β signaling pathway and tumor growth (shown in blue circle). In contrast, overexpression of either SOD2 or OGG1 suppresses oxidative stress-induced EBV DNA damage, thus preventing the HKDC1 interruption-induced suppression of EBV and tumor growth.

## Discussion

In this study, we found that the expression of LMP1 upregulated the expression of PGC1β through activation of NF-κB, with activated PGC1β upregulating the expression of OGG1 through coactivation of NRF1 and GABPα. We further found that a HKDC1 interruption-mediated generation of ROS triggered EBV DNA damage, resulting in LMP1-mediated suppression of the PGC1β/HKDC1/OGG1 signaling pathway, subsequently suppressing EBV-associated tumorigenesis.

In order to evaluate the gene expression in NKTCL cells, we selected T-lymphocytes from peripheral blood as the control. While T-lymphocytes were relatively few, they were sufficient for mRNA analysis, but not enough for western blotting. In this case, we chose peripheral blood mononuclear cells (PBMCs) as the alternative control for gene analysis. Our results showed that the mRNA expression was not significantly different between T-lymphocytes and PBMCs, and thus PBMCs were finally chosen as a control throughout this study. Our results showed that the Tf-D-HKC8 peptide blocked the binding of HKDC1 with VDAC1, triggering the release of mitochondrial superoxide, resulting in subsequent oxidative stress. In order to specifically block the Tf-D-HKC8 peptide-mediated mitochondrial oxidative stress, we used the SOD2-expressing lentivirus as a ROS scavenger throughout this study. The SOD2 protein is known to convert mitochondrial superoxide into the less toxic hydrogen peroxide, and unlike other ROS scavengers, such as SOD1, which are mostly located in the cytosol, SOD2 is also found in the mitochondria. In addition, catalase, which is known to detoxify hydrogen peroxide, has little effect on mitochondrial superoxide, while N-acetyl cysteine (NAC), which is a general antioxidant, can only transiently scavenge a certain amount of ROS and cannot kinetically scavenge ROS when shortly oxidized. Based on these observations, the SOD2 mitochondrial ROS scavenger was the best choice for this study, as previously mentioned [37, 38].

Our results showed that the expression of LMP1 and PGC1β was significantly increased in different NKTCL cells compared with healthy PBMCs. Additionally, we found that infection with LMP1-expressing lentivirus significantly increased the PGC1β luciferase reporter activity, with the LMP1-responsive element on the PGC1β promoter being identified as the one for the NF-κB transcription factor. Accordingly, the expression of LMP1 was significantly increased in healthy PBMCs, whereas knocking-down LMP1 in NKTCL cells significantly decreased the ability of NF-κB p65 to bind to the PGC1β promoter. Furthermore, we noted that a NF-κB knockdown of either subunit p65 or p50 significantly decreased the expression of PGC1β. These results provided very powerful evidence for the upregulation of PGC1β by EBV LMP1 through the activation of NF-κB. Many studies have reported the LMP1-mediated oncogenicity through the promotion of glycolysis and activation of NF-κB [12–17, 39], the detailed mechanism remains largely unknown. This study provided a bridge linking the mechanisms of the activation of LMP1/NF-κB and tumor development through the activation of PGC1β. In addition, our preliminary data showed that many EBV-associated tumors with a highly upregulated expression of PGC1β, did not have active LMP1 protein. We assumed that transient exposure to LMP1 might trigger epigenetic changes on the PGC1β promoter, resulting in the long-lasting upregulation of PGC1βduring the subsequent latent stage of LMP1 [40]. Therefore, targeting PGC1β might become more attractive for tumor development than targeting LMP1 in the latent stage and we are currently investigating this [41].

Our study showed that the expression of PGC1β and OGG1 was significantly increased in different NKTCL cells compared with healthy PBMCs. Additionally, we found that the expression of PGC1β significantly increased the OGG1 luciferase reporter activity, and identified the NRF1 and GABPα binding sites as the PGC1β-responsive elements on the OGG1 promoter. The expression of PGC1β was significantly increased in healthy PBMCs, whereas knocking-down PGC1β significantly decreased the binding ability of both NRF1 and GABPα to the PGC1β promoter. As knockdown of either NRF1 or GABPα significantly decreased the expression of OGG1, we concluded that its expression was regulated by PGC1β through the coactivation of NRF1 and GABPα. The expression of OGG1 was shown to minimize the ROS-mediated DNA damage, which is very important for the survival of EBV DNA, as it is relatively naked compared with histone-wrapped somatic DNA, making it more susceptible to attacks by ROS, especially during the replication process. In case that the ROS-mediated base incision cannot be repaired in time, the EBV DNA will eventually be destructed or lose its potency [35, 42–44]. However, the PGC1β expression-mediated upregulation of OGG1 was demonstrated to protect the EBV genome from ROS-mediated DNA damage, favoring EBV DNA survival from the mitochondrial dysfunction-mediated generation of ROS. Additionally, we recently found that PGC1β promoted glycolysis metabolism through the activation of LDHA [25]. Furthermore, it was shown to upregulate the expression of HKDC1 through the activation of SREBP1, resulting in increased mitochondrial function and glucose metabolism, thus favoring tumor growth [26]. In addition, we also found that PGC1β promoted mitochondrial biogenesis and glycolysis metabolism [20–23]. Taken together, we concluded that the LMP1-mediated expression of PGC1β promoted EBV-mediated tumorigenesis, in particular by favoring EBV DNA survival through the activation of OGG1.

We further showed that interruption of HKDC1 by either shHKDC1 or Tf-D-HKC8 peptide [36] led to the decreased interaction of HKDC11 with VDAC1 [45], triggering mitochondrial dysfunction, generation of ROS, and ROS-mediated DNA damage. Surprisingly, the interruption of HKDC1 was also shown to inhibit the expression of LMP1 and its downstream target genes, including PGC1β, OGG1, and HKDC1 itself as well as EBV-related proteins, including Zta, EA-D, and P-gp [6, 7, 46, 47]. However, we observed that overexpression of SOD2 and OGG1 reversed this effect completely and partly, respectively, indicating that the HKDC1 interruption-mediated suppression of EBV and target genes was primarily due to the generation of ROS and ROS-mediated EBV DNA damage. Taken together, we demonstrated that the HKDC1 interruption-mediated generation of ROS suppressed the EBV genome through the induction of DNA damage, resulting in the suppression of LMP1 and its target genes, including PGC1β, OGG1, and HKDC1 itself. Consequently, the suppression of HKDC1 was shown to trigger further mitochondrial dysfunction and generation of ROS, providing a positive feedforward loop for the generation of ROS and subsequent suppression of the EBV genome.

Our results showed that interruption of HKDC1 by the Tf-D-HKC8 peptide [36] in NKTCL cells significantly suppressed the cellular deoxyglucose uptake, colony formation, cell migration and invasion, and cell proliferation in vitro. Furthermore, we observed that interruption of HKDC1 by shHKDC1 significantly suppressed in vivo tumor formation and increased mouse survival; overexpression of SOD2 completely, whereas OGG1 only partly, reversed this effect, suggesting that the HKDC1 interruption-mediated antitumor effect was due to the generation of ROS and subsequent ROS-mediated DNA damage. Furthermore, our previous studies showed that the level of the expression of HKDC1 was significantly increased in NKTCL cells, whereas that was not the case in healthy PBMCs, which exhibited very low levels [26, 36]. These findings make HKDC1 a very attractive target for the development of anti-EBV and antitumor drugs, as interruption of HKDC1 was shown to not significantly affect normal cells. As our data indicated that the EBV genome seems to be a potential driving force for tumorigenesis in NKTCL cells through the LMP1-mediated PGC1β/HKDC1/OGG1 signaling pathway [26, 32, 33] and targeting HKDC1 is especially efficient in NKTCL cells, the development of novel molecules targeting HKDC1 is currently under investigation.

## Conclusions

LMP1 is the potential driving force for EBV-mediated tumorigenesis through activation of the PGC1β/HKDC1/OGG1 signaling pathway. Furthermore, HKDC1 interruption-mediated mitochondrial dysfunction forms a positive feed forward loop for ROS outbreak, subsequently suppressing EBV replication and LMP1/PGC1β signaling pathway-mediated tumor growth. Taken altogether, this study provides a novel therapeutic strategy for targeting HKDC1 for NKTCL treatment.

## Supplementary Information

ESM 1 (DOCX 20.3 MB)

## Abbreviations

EBV: Epstein-Barr virus
GABPα: GA-binding protein α (NRF2)
HKDC1: Hexokinase domain component 1
LMP1: latent membrane protein 1
MMP: mitochondrial membrane potential (ΔΨm)
NRF1: nuclear respiratory factor 1
NF-κB: nuclear factor-κB
NKTCL: Natural killer/T-cell lymphoma
OGG1: 8-oxoguanine DNA glycosylase 1
O_2_.^−^: superoxide anions
PGC1β: peroxisome proliferator-activated receptor-γ (PPARγ) coactivator-1β
P-gp: P-glycoprotein
ROS: reactive oxygen species
SOD2: superoxide dismutase 2
VDAC1: vascular endothelial growth factor 1
