# Correction to: Latent Membrane Protein 1 Promotes Tumorigenesis Through Upregulation of PGC1β Signaling Pathway

**DOI:** 10.1007/s12015-021-10160-8

**Published:** 2021-04-08

**Authors:** Jia Feng, Qi Chen, Ping Zhang, Xiaodong Huang, Weiguo Xie, Hongyu Zhang, Paul Yao

**Affiliations:** 1 Department of Hematology, Peking University Shenzhen Hospital, 518036 Shenzhen, People’s Republic of China; 2 Institute of Rehabilitation Center, Tongren Hospital of Wuhan University, 430060 Wuhan, People’s Republic of China

**Correction to: Stem Cell Rev and Rep.**

10.1007/s12015-020-10112-8

The above original version of article was published online on Jan 9^th^, 2021, while unfortunately, all the authors claim that this article contained 2 non-intentional editing mistakes.

Figure 5e/5 h (panel 1 and 4), and Fig. 6f (panel 1 and 3) were found to be misplaced after careful examination. Here below was what happened during our ppt slide preparation.

We imported 7–9 of representative pictures or group pictures (blue, green and merge, for Fig. 5e) for each treatment in each ppt slide, then chose and copied one series of representative pictures or group pictures from each treatment, and then pasted them in a new slide to assemble all 4 treatments together, while during this process, we removed the correct panel, but pasted the wrong pictures to the first panel by mistake. This error was happened because all the pictures lost their original name labeling when they were imported into ppt slides, and the operator got sometimes confused to identify so many pictures in one ppt slides during editing.

We apologize that Fig. 5e/5 h and Fig. 6f were indeed our careless editing mistakes, while we would like to emphasize that this is a non-intentional error that created by authors during the process of manuscript preparation. The correct Fig. 5 and 6 were shown below.





