# Supplementary material for: Latent Membrane Protein 1 Promotes Tumorigenesis Through Upregulation of PGC1β Signaling Pathway
Source: Stem Cell Rev Rep. 2021 Jan 9;17(4):1486–99. doi: 10.1007/s12015-020-10112-8 (PMC8316210; doi:10.1007/s12015-020-10112-8)
Supplement: Supplementary file 1 — (DOCX 20.3 MB) [file 12015_2020_10112_MOESM1_ESM.docx]

**Latent Membrane Protein 1 Promotes Tumorigenesis through Upregulation of PGC1β Signaling Pathway**

Jia Feng^1,^*, Qi Chen^1,^*, Ping Zhang^1^, Xiaodong Huang^2^, Weiguo Xie^2^,

Hongyu Zhang^1,#^, Paul Yao^1,#^

**Supplementary Materials**

**Data S1: Materials and Methods**

**Reagents and materials**. Written consent was obtained from participants and peripheral blood mononuclear cells (PBMC) from either healthy or NKTCL patients were isolated from peripheral blood using Lymphoprep^TM^ reagents (#07861). The human NK cells were isolated from healthy PBMC cells using EasySep™ Human NK Cell Isolation Kit from Stem Cell Technologies. Some of the PBMCs were conditionally immortalized using a hTERT lentivirus vector with an extended life span to achieve a higher transfection efficiency and experimental stability (1, 2). The NKTCL cell lines, including HANK1, NK92, SNT8 and SNK6 cells, were purchased from ATCC and cultured in RPMI 1640 medium containing 2mmol/l glutamine supplemented with 100 U/ml penicillin, 100 μg/ml streptomycin, 10% human serum and 1000 U/ml recombinant human IL-2. All cells were maintained in a humidified incubator with 5% CO_2_ at 37°C.

Antibodies for AP2α (sc-12726), β-actin (sc-47778), C/EBPε (sc-130029), EBV Ea-D (sc-58121), EBV ZEBRA (BZ1, sc-53904), GABPα (sc-28312), Ki-67 (sc-101861), NF1 (sc-74445), NFκB p65 (sc-398442), NRF1 (sc-101102), Oct1 (sc-8024), RXRβ (sc-742), SOD2 (sc-30080), Sp1 (sc-17824), SREBP1 (sc-13551), and VDAC1 (sc-390996) were obtained from Santa Cruz Biotechnology. Antibodies for H2AX (ab20669), γH2AX (ab2893), PGC1α (ab54481), PGC1β (ab240188) and P-gp (ab129450) were obtained from Abcam. The antibodies for AP1 (NBP1-89544), HKDC1 (NBP1-82108), EBV LMP1 (NBP1-79009), OGG1 (NB100-106) and 8-oxo-dG (4354-MC-050) were obtained from Novus Biologicals. 3-nitrotyrosine (3-NT) was measured using the 3-Nitrotyrosine ELISA Kit (ab116691 from Abcam), and NFκB activation was evaluated using NFκB p65 Transcription Factor Assay Kit (ab133112 from Abcam). Nuclear extracts were prepared using the NE-PER Nuclear and Cytoplasmic Extraction Reagents Kit (Pierce Biotechnology). Epstein-Barr virus–encoded RNA (EBER) in situ hybridization for both cells and tissues was conducted using ZhongShan ISH-5022 In Situ Hybridization Kit (#ISH5022 from ZSGB, Beijing) per manufacturers’ instruction. Protein concentration was measured using the Coomassie Protein Assay Kit (Pierce Biotechnology) per manufacturers’ instructions. The siRNA for GABPα, NFκB p65 or p50, NRF1, SREBP1 and negative control (#AM4636) were purchased from Ambion. The Lipofectamine™ Reagent (Invitrogen) was used for DNA transfection (3).

**Construction of human PGC1β/OGG1 reporter plasmid**. In order to construct PGC1β/OGG1 reporter plasmids, the PGC1β/OGG1 gene promoters (2kb upstream of the transcription start site plus first exon) were amplified from Ensembl gene ID: PPARGC1B-201 ENST00000309241.9. (for PGC1β) and OGG1-201 ENST00000302003.11 (for OGG1) by PCR from human genomic DNA and subcloned into the pGL3-basic vector (#E1751, Promega) using the restriction sites of Mlu I and Xho I with the following primers: PGC1β forward: 5’-gcgc-acgcgt- aca gaa ccc agg act cct aac -3’ (Mlu I) and PGC1β reverse: 5’- atcg- ctcgag- ctg cgt gtc agc gag ata gtt -3’ (Xho I); OGG1 forward: 5’-gcgc-acgcgt- tca aac tgg ttt gta ttt att-3’ (Mlu I) and OGG1 reverse: 5’- atcg- cgg aaa gat tgt cca gaa ggc -3’ (Xho I); To map PGC1β or OGG1 promoter activity, the related deletion promoter constructs were generated by PCR methods and subcloned into the pGL3-basic vector (4).

**Preparation of knockdown lentivirus.** The lentivirus for stable knockdown of either HKDC1 or PGC1β was prepared previously in our lab (3, 5). The stable knockdown cells for GABPα and OGG1 and related non-target control (CTL) were prepared through infection of tumor cell lines (e.g. SNK6) using shRNA lentivirus particles from Santa Cruz Biotech for human GABPα (sc-37100-V), OGG1 (sc-43983-V) or non-target control (sc-108080). The positive knockdown cells were selected using 10μg/ml of puromycin, and the stable knockdown cell line was confirmed by real time PCR based on an mRNA decrease of more than 65% compared to the control group (see primers in Table S1).

**Preparation of expression lentivirus**. The expression lentivirus for human HKDC1, PGC1β and SOD2 were generated in our lab as described previously (3, 5, 6). In order to prepare expression constructs, the EBV LMP1, human GABPα and OGG1 were amplified from full length cDNA of EBV LMP1 (Plasmid #36955 from Addgene), GABPα and OGG1 (obtained from Open Biosystems) and subcloned into either pLVX-Puro vector or pLVX-IRES-Hyg vector (from Clontech) using the following primers with the introduction of underlined restriction sites: LMP1 forward primer: 5’- atcg- ggatcc- atg gaa cac gac ctt gag agg - 3’ (BamH I); and LMP1 reverse primer: 5’- gtac- tctaga-tta gtc ata gta gct tag ctg -3’ (Xba1); GABPα forward primer: 5’- atcg- ctcgag- atg act aaa aga gaa gca gag -3’ (Xho I); and GABPα reverse primer: 5’- gtac- tctaga- tca att atc ctt ttc cgt ttg -3’ (Xba1); OGG1 forward primer: 5’- atcg- ggatcc- atg cct gcc cgc gcg ctt ctg - 3’ (BamH I); and OGG1 reverse primer: 5’- gtac- tctaga- cca tct agc ctt ccg gcc ctt -3’ (Xba1); The virus for HKDC1, PGC1β, EBV LMP1, GABPα and OGG1 or empty control (CTL) was expressed by Lenti-X™ Lentiviral Expression Systems (from Clontech), and this lentivirus was used for generation of stable tumor cell lines for overexpression using either puromycin or hygromycin as selection antibiotics (3, 5).

**Peptide synthesis and preparation**. The HKDC1-based peptides were synthesized by GL Biochem (Shanghai, China) with a level of > 85% purity. The peptides were dissolved in DMSO (dimethyl sulfoxide) and stored in aliquots at -20°C. Peptide concentration was determined by UV spectrophotometry at 280nm and calculated based on amino acid composition. The final concentration of DMSO used in this study was controlled to be no more than 0.5%. Detailed information on the synthesized peptides include Tf-D-Scram (**HAIYPRH**-*EILNKEKK*) and Tf-D-HKC8 (**HAIYPRH**-*LQQAQKEN*) as described previously in our lab (7). The cell-penetrating sequence is underlined, the HKDC1-targeting sequences are in italics, and amino acids in the D-configuration are marked in bold (8).

**Hexokinase (HK) activity assay.** Total HK activity from cell lysates was measured as the glucose phosphorylating capacity of whole cell extracts using a standard G6PDH-coupled assay (9, 10). The glucose and ATP-dependent reduction of NADP was monitored by a 96-well microplate reader at 340nm in the presence of excess G6PDH. All assays (final assay mixture composition: 1 U/ml G6PDH, 0.5mg/ml NADP, 6.7mM ATP, 7.7mM MgCl2, 4.0mM Glucose, 45mM KCl, 1mM NaH2PO4, 10.6mM monothioglycerol, 0.01% Triton X-100, 0.5mM EDTA, and 42 mM Tris HCl, pH 8.5) were performed at 25°C under conditions of linear HK-limited NADPH formation. Total HK activity was normalized for cellular protein content and expressed in enzyme activity units corresponding to the glucose phosphorylation rate in micromoles per minute (11).

**RT reaction and real-time quantitative PCR.** Total RNA from treated cells was extracted using the RNeasy Micro Kit (Qiagen), and the RNA was reverse transcribed using an Omniscript RT kit (Qiagen). All the primers were designed using Primer 3 Plus software with the Tm at 60°C, primer size of 21bp, and product length in the range of 140-160bp (see Table S1). The primers were validated with an amplification efficiency in the range of 1.9-2.1, and the amplified products were confirmed with agarose gel. Real-time quantitative PCR was run on iCycler iQ (Bio-Rad) using the Quantitect SYBR green PCR kit (Qiagen). The PCR was performed by denaturing at 95°C for 8 min followed by 45 cycles of denaturation at 95°C, annealing at 60°C, and extension at 72°C for 10s, respectively. 1 µl of each cDNA was used to measure target genes. β-actin was used as the housekeeping gene for transcript normalization, and the mean values were used to calculate relative transcript levels with the ^ΔΔ^CT method per instructions from Qiagen. In brief, the amplified transcripts were quantified by the comparative threshold cycle method using β-actin as a normalizer. Fold changes in gene mRNA expression were calculated as 2^−ΔΔCT^ with CT = threshold cycle, ΔCT=CT (target gene)-CT(β-actin), and the ΔΔCT =ΔCT (experimental)-ΔCT (reference) (4, 12).

**Luciferase reporter assay.** 1.0×10^5^ of cells were seeded in a 6-well plate with complete medium to grow until they reached 80% confluence. Cells were then cotransfected by 3µg of reporter constructs, together with 0.2µg of pRL-CMV-Luc *Renilla* plasmid (from Promega). The treated cells were then harvested and the luciferase activity assays were carried out using the Dual-Luciferase^TM^ Assay System (Promega). The transfection efficiencies were normalized using a cotransfected *Renilla* plasmid according to manufacturers’ instructions and the reporter activities for PGC1β and OGG1 were calculated (4).

**Chromatin immunoprecipitation (ChIP).** Cells were washed and crosslinked using 1% formaldehyde for 20 min and terminated by 0.1M glycine. Cell lysates were sonicated and centrifuged. 500µg of protein were pre-cleared by BSA/salmon sperm DNA with preimmune IgG and a slurry of Protein A Agarose beads. Immunoprecipitations were performed with the indicated antibodies, BSA/salmon sperm DNA and a 50% slurry of Protein A agarose beads. Input and immunoprecipitates were washed and eluted, then incubated with 0.2mg/ml Proteinase K for 2h at 42˚C, followed by 6h at 65˚C to reverse the formaldehyde crosslinking. DNA fragments were recovered through phenol/chloroform extraction and ethanol precipitation. A ~150bp fragment on the related promoter was amplified by real-time PCR (qPCR) using the primers provided in Table S1 (4, 12).

**Immunoprecipitation (IP) and western blotting (WB).** Cell lysates were pre-cleared by pre-immune IgG plus Protein A agarose beads for 2 hours and the supernatants were immunoprecipitated by the indicated antibodies and a 50% slurry of Protein A Agarose beads overnight at 4˚C (13). After washing with buffer containing 50 mM Tris, pH 7.5, 150 mM NaCl, 1% NP-40, and 0.5% deoxycholate with protease inhibitors, proteins were released, separated on 10% SDS-PAGE gels, blotted by primary antibodies, and then simultaneously incubated with the differentially labeled species-specific secondary antibodies, anti-RABBIT IRDye™ 800CW (green) and anti-MOUSE (or goat) ALEXA680 (red). Membranes were scanned and quantitated by the ODYSSEY Infrared Imaging System (LI-COR, NE) (14).

**Immunostaining**. The treated SNK6 cells were transferred to cover slips coated with 0.1% gelatin, fixed by 3.7% formaldehyde at 37ºC for 15 min, permeabilized by 1% BSA+0.2% Triton X-100 in PBS for 1 hour, and then blotted with 40μg/ml (dilute 1:50) of either Ki-67 (MIB-1) or 8-oxo-dG monoclonal antibodies for 2 hours. The cells were then washed three times and the FITC labeled anti-mouse/rabbit secondary antibody (1:100) was added for blotting for another 1 hour. After thorough washing, the slides were visualized and photographed, the nuclei of cells were stained with 4’,6-diamidino-2-phenylindole dihydrochloride (DAPI, #D9542, from Sigma), and the staining of 8-oxo-dG or positive Ki-67 cells was quantitated.

**[^3^H]-deoxyglucose uptake.** 1x106 treated cells were suspended and rinsed with PBS three times and were then incubated with 1ml of PBS containing 1.0uCi 3H-deoxyglucose for 5 min at 37°C. Cells were washed with cold PBS 3 times and solubilized in 1ml of 1M NaOH for 60 min at 37°C. They were then neutralized with an equal volume of 1 M HCl and counted in 10 ml scintillation mixture, and the final results were normalized by protein level.

**Measurement of ROS generation.** Treated cells were seeded in a 24-well plate and incubated with 10μM CM-H2DCFDA (Invitrogen) for 45 min at 37°C, and then the intracellular formation of reactive oxygen species (ROS) was measured at excitation/emission wavelengths of 485/530nm using a FLx800 microplate fluorescence reader (Bio-Tek). The data was normalized as arbitrary units (4, 15).

**Measurement of DNA breaks**. 8-OHdG formation was measured using an OxiSelect™ Oxidative DNA Damage ELISA Kit (Cat No. STA320, from Cell Biolabs Inc.) per manufacturers’ instructions. The formation of γH2AX was measured from nuclear extracts by western blotting using H2AX as the input control (4).

**Measurement of apoptosis and cell death.** Apoptosis was evaluated by TUNEL assay using the In Situ Cell Death Detection Kit™ (Roche). Cells were fixed in 4% paraformaldehyde and labeled with TUNEL reagents. Stained cells were photographed by a fluorescence microscope and further quantified by FACS analysis (15). For cell death analysis, the propidium iodide (PI) staining was performed by the addition of 6.25 µg/ml of PI to the cells, and cell death was immediately evaluated by FACS analysis (8). Different concentrations of peptides were used for treatment of either adhesion (12 hours) or suspension (6 hours) cells, and the half-maximal cell death activity (IC_50_) values (µM) were then calculated based on the cell death data obtained from PI/FCAS analysis (8).

**Measurement of mitochondrial function.** Intracellular ATP level was determined using the luciferin/luciferase-induced bioluminescence system. An ATP standard curve was generated at concentrations of 10^-12^-10^-3^M, and intracellular ATP levels were calculated and expressed as nmol/mg protein. Mitochondrial membrane potential (Δψm) was measured using TMRE (from Molecular Probes T-669) staining. A 600μM T-669 stock solution was prepared using DMSO. Cells were grown on coverslips and immersed in 600nM TMRE for 20 min at 37°C to load them with dye. The labeling medium was then aspirated and the cells were immersed in 150nM TMRE to maintain an equal distribution of the fluorophore. The coverslips were mounted with live cells onto confocal microscopes to image the cells using 548nm excitation/573nm emission filters, and the intensity of TMRE fluorescence was measured using Image J software. Data from 10-20 cells were collected for each experimental condition and mean values of fluorescence intensity ± SEM were calculated (2).

**Detection of EBV copy number.** Genomic DNA was extracted from treated SNK-6 cells or SNK-6 tumor tissue in mice using a QIAamp DNA Mini Kit (Qiagen). The EBV DNA copy number was measured through qPCR using 50ng of total DNA with EBV BMRF1 primers (see Table 1), and the results were normalized using cellular β-actin (primers see Table 1) as an internal control (16, 17). The Namalwa cell line, which contains 2 EBV viral genome copies, was used as a standard to prepare calibration curves for both EBV BMRF1 and β-actin genes, and the EBV viral load was presented as the number of viral genomes per cell (18, 19).

**DNA synthesis by [^3^H]-thymidine incorporation**. Cell proliferation was evaluated as the rate of DNA synthesis by [^3^H]-methylthymidine incorporation (20). Cells were pooled in 24-well plates until they reached 80% confluence and then the indicated chemicals were added and incubated for 24 hours. At the end of the treatment, cells were incubated with serum-free media containing ^3^H-methylthymidine (0.5 µCi/well) for 2 hours and then washed twice with PBS. Cellular DNA was precipitated using 10% trichloroacetic acid and solubilized with 0.4M NaOH (0.5 ml/well). Incorporation of ^3^H-methylthymidine into the DNA was measured in a scintillation counter and was determined as counts per minute (CPM) (4).

**Colony formation in soft agar**. This assay is a method for evaluating the ability of individual cell lines to grow in an anchorage-independent manner. Cells were resuspended in DMEM containing 5% FBS with 0.3% agarose and layered on top of 0.5% agarose in DMEM on 60-mm plates. 1000 cells were seeded in 60mm soft agar dishes for 30 days. The dishes were examined twice per week, and colonies that grew beyond 50mm in diameter were scored as positive. Each experiment was done in quadruplicate (4).

**Comet Assay**. The comet assay was conducted using the Comet Assay Kit (# 4250-050-k from Trevigen) per manufacturers’ instructions. In brief, treated cells were combined with comet agarose at 37°C and transferred onto agarose-coated slides. The electrophoresis was performed (electric current: 300 mA constant; 1 V/cm) after lysis in prechilled alkaline lysis buffer for 30-60 mins at 4°C and unwinding of the DNA in alkaline solution for 30min at 4°C in dark conditions. The slides were neutralized in salt buffer (pH 7.5) and stained with SYRB Green I dye. Following two cycles of rinsing and replacing with 70% ethanol for 5 mins, the slides were air dried before the comets were evaluated by fluorescence microscopy and the lengths of the tails were quantitated.

**Migration and invasion assays**. Cell migration and invasion assays were performed in 24-well chemotaxis plates with an 8μm polycarbonate filter membrane. The plates were coated with 20μg Matrigel for invasion assays and uncoated for migration assays. Invasion and migration were expressed as the number of migrated cells bound per microscopic field and averaged from at least four fields per assay in at least 4 experiments (21).

**In vivo mouse experiments**. Balb/c athymic nude male mice (6 weeks old) were obtained from the Guangdong Medical Animal Center. All procedures involving mice were conducted in accordance with NIH regulations concerning the use and care of experimental animals and were approved by the Institutional Animal Care and Use Committee of Peking University Shenzhen Hospital. The 2x10^6^ viable treated tumor cells were washed, harvested in PBS, and then injected into the lateral tail vein in a volume of 0.1ml. Mice were monitored for changes in body weight and sacrificed when values fell below 20% of their initial weight, and the survival curve was calculated and the final tumor tissues were isolated for biomedical analysis. The lungs of sacrificed mice were isolated and fixed in 10% formalin. The number of surface metastases per lung was determined under a dissecting microscope. Parts of the tumor tissues were fixed in 4% buffered formaldehyde, paraffin embedded, and sectioned to 4mm thickness. They were then either processed for immunohistochemistry (IHC) or histopathological analyses, which were performed with H&E staining. Images were taken using a Carl Zeiss MIRAX MIDI slide scanner, and analyses were performed using a 3DHISTECH Pannoramic Viewer. Parts of the tumor tissues were isolated for *in vivo* monitoring of superoxide anion release, gene expression was measured through real time PCR for mRNA and Western Blotting for protein levels, the copies of the EBV genome were measured by real time PCR, and the binding of HKDC1 and VDAC1 was evaluated by IP/WB (3, 4, 22).

**Immunohistochemistry (IHC).** The tumor tissue slides were first fixed in 3.7% formaldehyde solution, permeabilized by 1% BSA+0.2% Triton X-100 in PBS, and then blotted by 40μg/ml of antibodies for 8-oxo-dG for 2 hours. After another 1 hour of blotting by FITC labeled anti-mouse/rabbit secondary antibodies, the slides were visualized and photographed, and the protein expression (60 cells in each group) were quantitated by Image J. software (3).

**In vivo superoxide release analysis.** Superoxide anion (O_2_^.-^) release from tumor tissues was determined using a luminol-EDTA-Fe enhanced chemiluminescence (CL) system supplemented with DMSO-TBAC (Dimethyl sulfoxide-tetrabutyl-ammonium chloride) solution for extraction of released O_2_^.-^ from tissues, as described previously (15). Superoxide levels were calculated from the standard curve generated by the xanthine/xanthine oxidase reaction (4).

**Statistical analysis.** The data was given as mean ± SEM; all of the experiments were performed at least in quadruplicate unless otherwise indicated. The unpaired Student’s t-tests or one-way analysis of variance (ANOVA) followed by the Turkey−Kramer test was used to determine statistical significance of different groups, and the two-way ANOVA followed by the Bonferroni post hoc test was used to determine the differences of two factors. The mouse survival curve was determined through Kaplan-Meier survival analysis using SPSS 22 software, and a *P* value < 0.05 was considered significant (4).

REFERENCES

1. Bodnar AG, Ouellette M, Frolkis M, Holt SE, Chiu CP, Morin GB, et al. Extension of life-span by introduction of telomerase into normal human cells. *Science.* 1998;279(5349):349-52.

2. Kong D, Zhan Y, Liu Z, Ding T, Li M, Yu H, et al. SIRT1-mediated ERbeta suppression in the endothelium contributes to vascular aging. *Aging Cell.* 2016;15(6):1092-102.

3. Zhang H, Li L, Chen Q, Li M, Feng J, Sun Y, et al. PGC1beta regulates multiple myeloma tumor growth through LDHA-mediated glycolytic metabolism. *Mol Oncol.* 2018;12(9):1579-95.

4. Zhang H, Li L, Li M, Huang X, Xie W, Xiang W, et al. Combination of betulinic acid and chidamide inhibits acute myeloid leukemia by suppression of the HIF1alpha pathway and generation of reactive oxygen species. *Oncotarget.* 2017;8(55):94743-58.

5. Chen X, Lv Y, Sun Y, Zhang H, Xie W, Zhong L, et al. PGC1beta Regulates Breast Tumor Growth and Metastasis by SREBP1-Mediated HKDC1 Expression. *Front Oncol.* 2019;9:290.

6. Wang X, Lu J, Xie W, Lu X, Liang Y, Li M, et al. Maternal diabetes induces autism-like behavior by hyperglycemia-mediated persistent oxidative stress and suppression of superoxide dismutase 2. *Proc Natl Acad Sci U S A.* 2019;116(47):23743-52.

7. Chen Q, Feng J, Wu J, Yu Z, Zhang W, Chen Y, et al. HKDC1 C-terminal based peptides inhibit extranodal natural killer/T-cell lymphoma by modulation of mitochondrial function and EBV suppression. *Leukemia.* 2020.

8. Shteinfer-Kuzmine A, Amsalem Z, Arif T, Zooravlov A, and Shoshan-Barmatz V. Selective induction of cancer cell death by VDAC1-based peptides and their potential use in cancer therapy. *Mol Oncol.* 2018;12(7):1077-103.

9. Bryson JM, Coy PE, Gottlob K, Hay N, and Robey RB. Increased hexokinase activity, of either ectopic or endogenous origin, protects renal epithelial cells against acute oxidant-induced cell death. *J Biol Chem.* 2002;277(13):11392-400.

10. Majewski N, Nogueira V, Bhaskar P, Coy PE, Skeen JE, Gottlob K, et al. Hexokinase-mitochondria interaction mediated by Akt is required to inhibit apoptosis in the presence or absence of Bax and Bak. *Mol Cell.* 2004;16(5):819-30.

11. Kroschewski H, Ortner S, Steipe B, Scheiner O, Wiedermann G, and Duchene M. Differences in substrate specificity and kinetic properties of the recombinant hexokinases HXK1 and HXK2 from Entamoeba histolytica. *Mol Biochem Parasitol.* 2000;105(1):71-80.

12. Zou Y, Lu Q, Zheng D, Chu Z, Liu Z, Chen H, et al. Prenatal levonorgestrel exposure induces autism-like behavior in offspring through ERbeta suppression in the amygdala. *Mol Autism.* 2017;8:46.

13. Metivier R, Penot G, Hubner MR, Reid G, Brand H, Kos M, et al. Estrogen receptor-alpha directs ordered, cyclical, and combinatorial recruitment of cofactors on a natural target promoter. *Cell.* 2003;115(6):751-63.

14. Ceradini DJ, Yao D, Grogan RH, Callaghan MJ, Edelstein D, Brownlee M, et al. Decreasing intracellular superoxide corrects defective ischemia-induced new vessel formation in diabetic mice. *J Biol Chem.* 2008;283(16):10930-8.

15. Yao D, Shi W, Gou Y, Zhou X, Yee Aw T, Zhou Y, et al. Fatty acid-mediated intracellular iron translocation: a synergistic mechanism of oxidative injury. *Free Radic Biol Med.* 2005;39(10):1385-98.

16. Verma D, Thompson J, and Swaminathan S. Spironolactone blocks Epstein-Barr virus production by inhibiting EBV SM protein function. *Proc Natl Acad Sci U S A.* 2016;113(13):3609-14.

17. Zuo L, Yu H, Liu L, Tang Y, Wu H, Yang J, et al. The copy number of Epstein-Barr virus latent genome correlates with the oncogenicity by the activation level of LMP1 and NF-kappaB. *Oncotarget.* 2015;6(38):41033-44.

18. Hui KF, and Chiang AK. Suberoylanilide hydroxamic acid induces viral lytic cycle in Epstein-Barr virus-positive epithelial malignancies and mediates enhanced cell death. *Int J Cancer.* 2010;126(10):2479-89.

19. Rose C, Green M, Webber S, Kingsley L, Day R, Watkins S, et al. Detection of Epstein-Barr virus genomes in peripheral blood B cells from solid-organ transplant recipients by fluorescence in situ hybridization. *J Clin Microbiol.* 2002;40(7):2533-44.

20. Somasundaram K, and El-Deiry WS. Inhibition of p53-mediated transactivation and cell cycle arrest by E1A through its p300/CBP-interacting region. *Oncogene.* 1997;14(9):1047-57.

21. Han HJ, Russo J, Kohwi Y, and Kohwi-Shigematsu T. SATB1 reprogrammes gene expression to promote breast tumour growth and metastasis. *Nature.* 2008;452(7184):187-93.

22. Zhang H, Lu J, Jiao Y, Chen Q, Li M, Wang Z, et al. Aspirin Inhibits Natural Killer/T-Cell Lymphoma by Modulation of VEGF Expression and Mitochondrial Function. *Front Oncol.* 2018;8:679.

**Table S1. Sequences of primers for the real time quantitative PCR (qPCR)**

| Gene | Species | Analysis | Forward primer (5'→3') | Reverse primer (5'→3') |
| --- | --- | --- | --- | --- |
| β-actin | Human | mRNA | gatgcagaaggagatcactgc | atactcctgcttgctgatcca |
| BZLF1 | EBV | mRNA | gggggataatggagtcaacat | tagcgtcccaaacataaatgc |
| BMRF1 | EBV | mRNA | tcctgtccaagtgctatgacc | gggagacctcgaagctgatta |
| LMP1 | EBV | mRNA | ccttggaattgtgctgttcat | tgagcaggatgaggtctagga |
| β-actin | Human | Genome | ctggacttcgagcaagagatg | aggaaggaaggctggaagagt |
| BMRF1 | EBV | Genome | ccgtcctgtccaagtgctat | gggagacctcgaagctgatta |
| HKDC1 | Human | mRNA | acgagtttgacctggacattg | ccatctcgatgttcctcatgt |
| GABPα | Human | mRNA | tttcttgggttggtgatgaag | tacaaatcatgtccccatcgt |
| PGC1β | Human | mRNA | tgctagcctcaccaaacactt | ttcttcctcttcctcctctgg |
| NFκB p65 | Human | mRNA | atcccatctttgacaatcgtg | gtcccgtgaaatacacctcaa |
| NRF1 | Human | mRNA | cgaggacacctcttacgatga | tcaaatacatgaggccgtttc |
| OGG1 | Human | mRNA | gatgttaccctggctcaactg | cgatgttgttgttggaggaac |
| ABCB1 | Human | mRNA | atagacagccgcagtcaagaa | gctactgtctttcctcgctca |
| SOD2 | Human | mRNA | gcctacgtgaacaacctgaac | tgaggtttgtccagaaaatgc |
| PGC1β | Human | ChIP | caccatgcctggctaatttt | agtggctcacgcctgtaatc |
| OGG1 | Human | ChIP | gcccagatggaactcgttag | gaggtaggaggcgccaat |
| HKDC1 | Human | ChIP | gagatgggaggatcacctgag | ggagcatttaccaatcccatt |

FIGURE S1

**Figure S1. Gene expression of healthy NK and PBMC cells.** Healthy NK and PBMC cells were isolated for mRNA analysis, n=4. Data were expressed as mean ± SEM.

FIGURE S2

**Figure S2. Representative pictures of full blots for Western Blotting.** (a) Representative full blots for Figure 1b. (b) Representative full blots for Figure 1e. (c). Representative full blots for Figure 1h.

FIGURE S3

**Figure S3. Representative pictures of full blots of Western Blotting for Figure 2h.**

FIGURE S4

**Figure S4. Representative pictures of full blots of Western Blotting for Figure 3i.**

FIGURE S5

**Figure S5. Representative pictures of full blots for Western Blotting.** (a) Representative full blots for Figure 4c. (b) Representative full blots for Figure 4d.

FIGURE S6

**Figure S6. Representative pictures of full blots for Western Blotting.** (a) Representative full blots for Figure 5d. (b) Representative full blots for Figure 5n.

FIGURE S7

**Figure S7. Representative pictures of full blots for Western Blotting.** (a) Representative full blots for Figure 7a. (b) Representative full blots for Figure 7c.
